# Volvulus of the stomach and wandering spleen after repair of congenital diaphragmatic hernia: unexpected manifestations in a neonate

**DOI:** 10.1186/s40792-022-01537-z

**Published:** 2022-09-23

**Authors:** Noboru Oyachi, Fuminori Numano, Tamami Fukatsu, Atsushi Nemoto, Atsushi Naito

**Affiliations:** 1 Department of Pediatric Surgery, Yamanashi Prefectural Central Hospital, 1-1-1 Kofu, Yamanashi, 409-8506 Japan; 2 Department of Neonatology, Yamanashi Prefectural Central Hospital, Kofu, Japan

**Keywords:** Congenital diaphragmatic hernia, Gastric volvulus, Splenic volvulus, Gastropexy, Infant

## Abstract

**Background:**

Congenital diaphragmatic hernia (CDH) is sometimes associated with complications involving herniation of intrathoracic organs, which further increase mortality rate. We encountered a case of postoperative gastric and splenic volvulus shortly after left CDH repair in a female neonate who was treated with gastropexy.

**Case presentation:**

At 39 weeks gestation, a female patient with left Bochdalek CDH was delivered (birth weight: 3748 g, Apgar score: 3/4). The patient was provided ventilator support with nitric oxide. After pulmonary hypertension improved, CDH repair was performed via the abdominal approach on day 7. The stomach, small intestine, large intestine, and spleen were herniated through a diaphragmatic defect of 4 × 2 cm. Although the diaphragm was directly closed, it was tight and the reconstructed diaphragm “dome” was shallow, restricting space for the spleen and stomach. Nonetheless, the spleen was positioned in the left upper abdomen and the stomach was positioned medially. The postoperative course was complicated by organo-axial gastric volvulus, and laparotomy was performed on day 14. In addition to the gastric volvulus, we confirmed a wandering splenic volvulus. The spleen was easily detorted and returned to the left upper abdomen. However, the patient experienced relapse of gastric volvulus without splenic volvulus. Gastropexy was performed electively on day 47. Postoperatively, the patient could be fed orally, and the patient’s development was satisfactory 6 years after surgery.

**Conclusions:**

The cause of these rare complications appeared to be tight direct diaphragmatic closure, which reduced space for the spleen and stomach beneath the left diaphragm.

## Background

Congenital diaphragmatic hernia (CDH) is sometimes associated with various complications involving herniation of intrathoracic organs, which further increase mortality rate. Among the serious intrathoracic gastrointestinal problems prior to CDH repair, intrathoracic gastric or splenic volvulus leading to gastric perforation or splenic infarction has been reported in a few CDH cases [1]. However, these complications rarely present after CDH repair [2–5].

We encountered a case of postoperative gastric and splenic volvulus shortly after left CDH repair in a female neonate who was treated with gastropexy.

## Case presentation

**Fig. 1:**
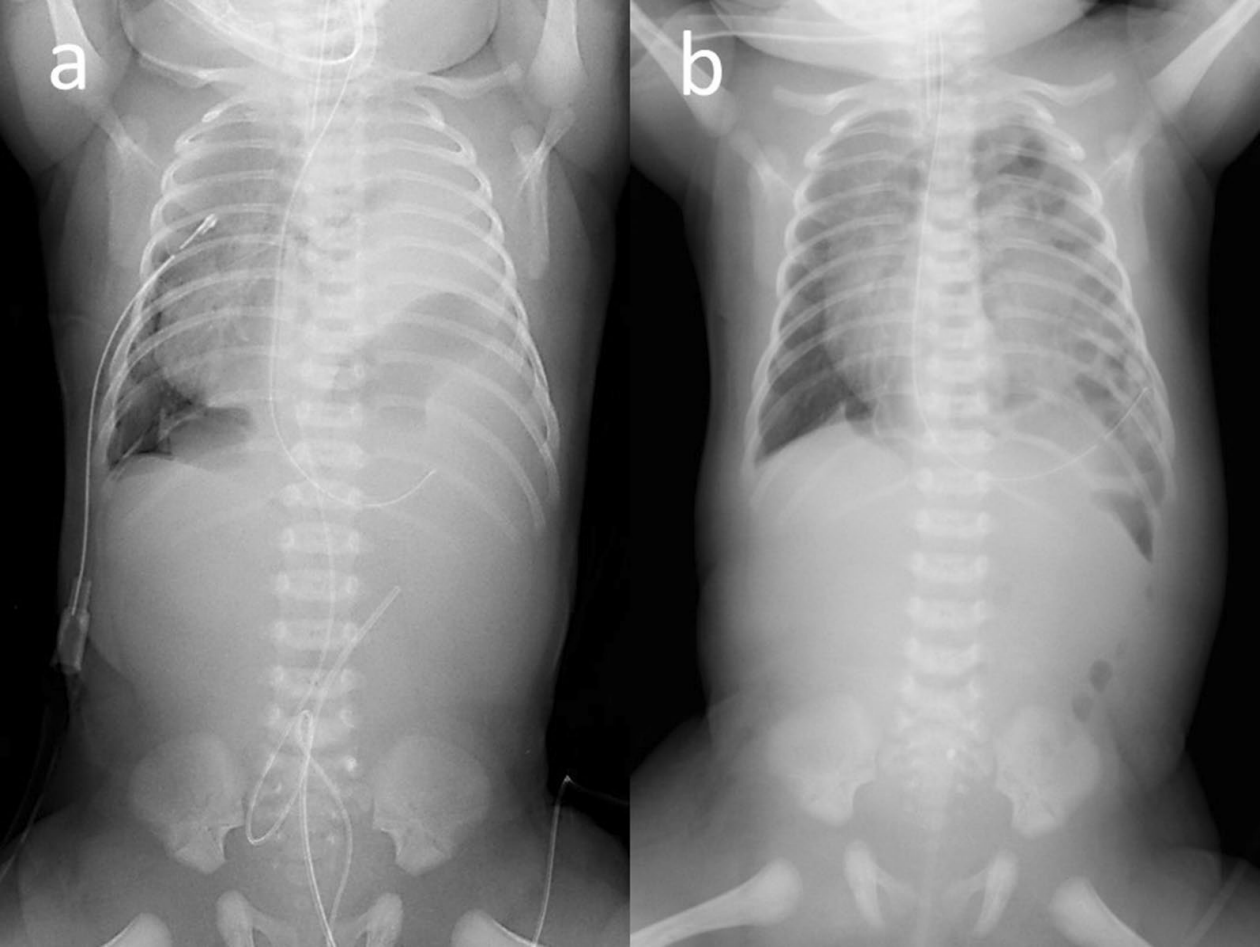
Abdominal and chest X-ray on admission (**a**) and day 4 (**b**). **a** Nitric oxide was inhaled and ventilator support was provided. A right pneumothorax was noted and required intrathoracic drainage until day 3. **b** The stomach, spleen, and small and large intestine were elevated in the thoracic cavity

At 39 weeks gestation, a female patient was delivered vaginally, with a birth weight of 3748 g and an Apgar score of 3/4. She was diagnosed with left Bochdalek CDH at 22 weeks gestation, and evaluation at 30 weeks gestation indicated moderate CDH without elevation of the liver, with an observed to expected lung area-to-head circumference ratio (o/e LHR) of 34.6%. The patient was immediately given ventilator support with nitric oxide. The patient demonstrated a right pneumothorax, and intrathoracic drainage was required until day 3 (Fig. 1). No other specific anomalies were recognized, and chromosomal abnormalities were not detected in routine karyotypes.

**Fig. 2:**
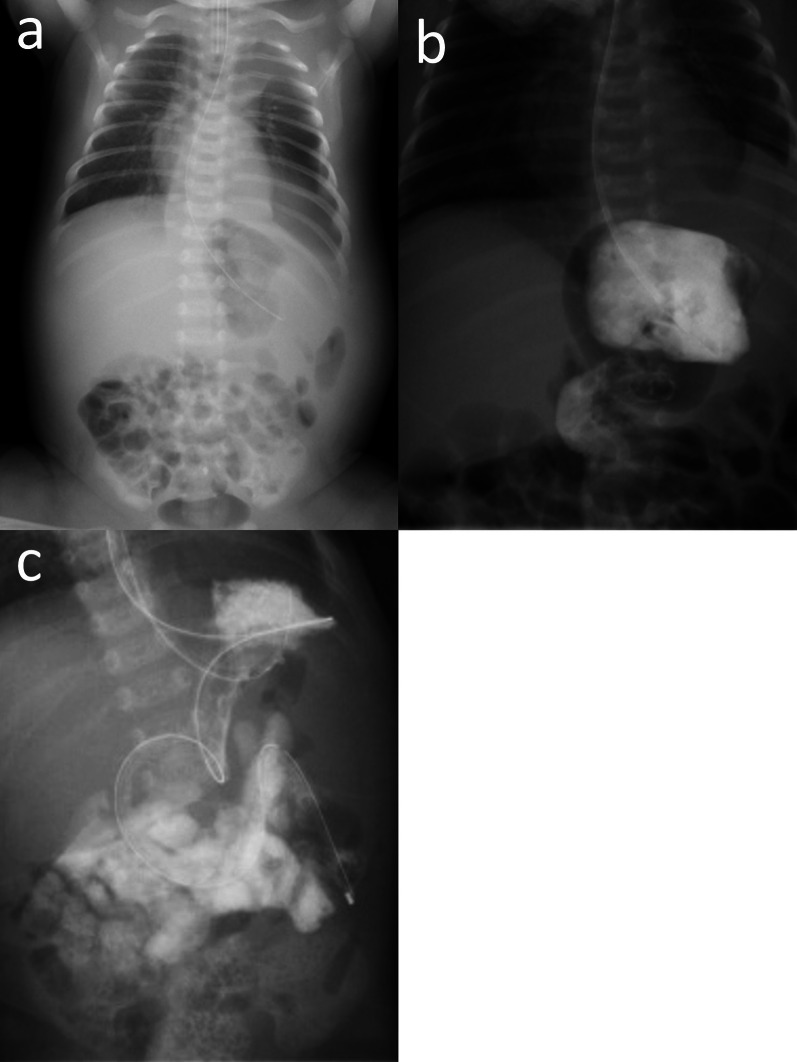
Abdominal X-ray (**a**) and upper gastrointestinal series (**b**, **c**). **a** Abdominal X-ray on postoperative day 1 demonstrated that the left diaphragm was pulled caudally, and the stomach was deviated medially. **b** Upper gastrointestinal series showed organo-axial gastric volvulus with the greater curvature crossing the stomach anteriorly. **c** The patient presented with recurrent gastric volvulus. In addition to gastric decompression, an enteral feeding tube was placed in the duodenum, and enteral feeding through the tube was initiated

After pulmonary hypertension improved, CDH repair was performed via the abdominal approach on day 7. The stomach, small intestine, large intestine, and spleen were herniated through a diaphragmatic defect of 4 × 2 cm without the posterior muscle rim. The defect was categorized as Defect C (a large (> 50%) portion of the chest wall devoid of diaphragm tissue) from the proposed defect diagram by the Congenital Diaphragmatic Hernia Study Group [6]. The intrathoracic hypermobilized spleen was large, with severe congestion. No splenic volvulus was observed, and we speculated that impaired venous return of the spleen was the cause of congestion. Direct closure of the diaphragm was chosen, which strained the diaphragm and caused the reconstructed diaphragm "dome" to be shallow, restricting adequate space for the spleen and stomach. Nonetheless, the spleen was positioned in the left upper abdomen and the stomach was positioned medially (Fig. 2a). The postoperative course was complicated by a severely dilated stomach and non-bilious vomiting, and the patient could not tolerate enteral feeding. The upper gastrointestinal series showed organo-axial gastric volvulus with the greater curvature crossing the stomach anteriorly (Fig. 2b). The management strategy was to place the infant on the right side or in the prone position after feeding, with the head elevated above the torso. Gastric tube decompression with a nasogastric tube was initiated, which temporarily improved the patient’s condition but was inadequate for long-term treatment.

On day 14, laparotomy was performed as there was no improvement with conservative therapy. In addition to organo-axial volvulus of the stomach, we confirmed a wandering spleen with splenic volvulus (torsion of 180°). The spleen was not infarcted and was easily detorted and returned to the left upper abdomen without splenopexy or gastropexy. However, the patient had a relapse of gastric volvulus, while ultrasound showed that the spleen was correctly located without volvulus. In addition to gastric decompression, an enteral feeding tube was placed in the duodenum and feeding through the tube was initiated (Fig. 2c). We selected a wait-and-see policy for conservative treatment.

However, the patient’s condition did not improve. Therefore, we elected to perform a gastric fixation on day 47. Organo-axial volvulus was identified, and the greater curvature of the stomach was adhered to the anterior abdominal wall. We performed gastropexy on the anterior abdominal wall at three points after dissecting and detorting the stomach. Splenopexy was not performed because the spleen was in the correct position without volvulus.

**Fig. 3:**
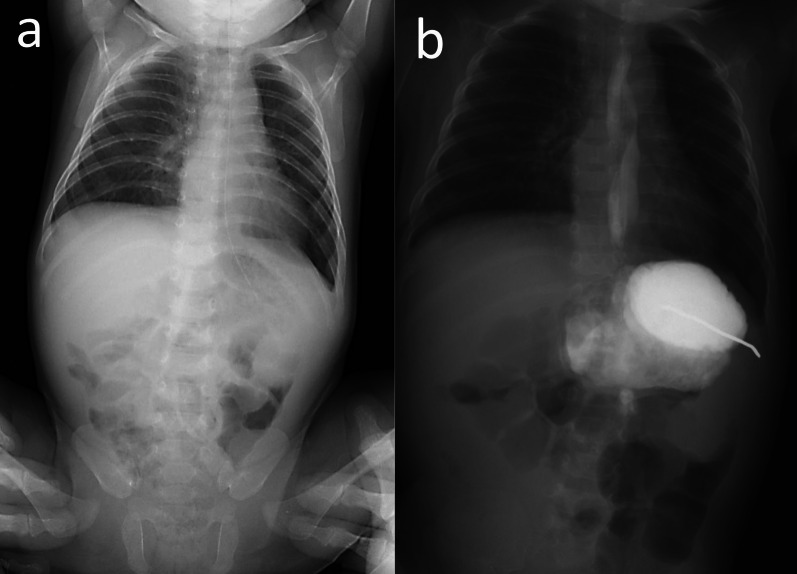
Postoperative X-ray (**a**) and upper gastrointestinal series (**b**). **a** Postoperatively, the stomach was correctly positioned without volvulus. **b** Gastroesophageal reflux persisted for a while

Postoperatively, the patient was fed orally and discharged on day 72 (Fig. 3). Gastroesophageal reflux persisted for a while, but the patient’s development was satisfactory 6 years after surgery.

## Discussion

Serious gastrointestinal diseases, such as intestinal perforation and intestinal torsion (including gastric and splenic volvulus), prior to diaphragmatic repair have been reported in several cases of CDH [1], and they cause catastrophic conditions [7]. However, in the present case, unexpected postoperative gastric and splenic volvulus were observed. Furthermore, even after the gastric and splenic volvulus were surgically detorted, the gastric volvulus recurred and required gastropexy.

The stomach is firmly held in place by the gastric ligament, esophageal hiatus, and retroperitoneal fixation of the duodenum. Gastric volvulus is defined as abdominal rotation of all or part of the stomach around one of its axes due to abnormalities, such as inadequate attachment or elongation of the gastric ligament [8]. In organo-axial gastric volvulus, which is the most common type and was observed in our case, the stomach rotates on its long axis, and the large curvature lies anteriorly. Gastric volvulus may occasionally occur acutely in older children, whereas chronic cases may be more common in neonates and infants [9]. In chronic cases in infancy, it has been noted that gastric volvulus can be released by repositioning or decompression of the stomach. Conservative treatment is often used, but some reports recommend early surgery in recurrent cases [8], and the treatment of chronic gastric volvulus depends on the symptoms [9, 10].

A wandering spleen is a rare disorder in which the ligamentous attachment to the stomach and the posterior abdominal wall is absent or weak, resulting in an abnormal spleen position [11]. Associated complications, such as splenic infarction, gastric volvulus, and intestinal obstruction, have been reported [2, 12]. The creation of a retroperitoneal pocket for splenopexy is considered useful for treatment. Although gastric and splenic volvulus each are rare malformations, multiple studies have shown that they often occur simultaneously because the stomach and spleen are in close proximity and share a few common ligaments.

Intrathoracic gastric and splenic volvulus have occasionally been reported in association with diaphragmatic hernia [1, 13]. Once the stomach or spleen has herniated into the thoracic cavity, the surrounding ligaments attached to them may be absent or lax, increasing the range of motion of the organs and causing volvulus [14, 15].

However, intra-abdominal volvulus of the stomach or spleen after diaphragmatic hernia repair is very rare [2–5]. In our case, it was unclear whether gastric volvulus occurred first, resulting in subsequent splenic volvulus or vice versa. At the time of CDH repair, as discussed in the case presentation, tight direct closure of the diaphragm reduced the space for the normal placement of the spleen and stomach beneath the left diaphragm. The spleen was placed in the left upper abdomen and the stomach was placed medially, but each organ appeared to deviate from its normal anatomical position. We presumed that the reduced space induced the rare complications of splenic torsion and gastric volvulus, which caused gastric obstruction immediately after diaphragmatic closure.

On the first re-opening of the abdomen, 180° volvulus of the spleen was also observed. The gastric volvulus could be resolved once splenic volvulus was detorted. The retroperitoneum was vulnerable; therefore, only splenic detorsion, without splenopexy or gastropexy, was performed. When the gastric volvulus recurred for the second time, there was no abnormality in the spleen. However, the stomach adhered to the surrounding tissues, which was thought to inhibit spontaneous resolution, and gastropexy was required. Considering the clinical course of this case, at the initial surgery patch closure to make the dome of the diaphragm, rather than tight direct closure, may have been a better choice, or gastropexy should have been performed at an earlier stage.

## Conclusions

We encountered a case of left-sided CDH complicated by prolonged gastric volvulus with gastric obstruction that was treated with gastropexy, following CDH repair. The cause of these rare complications appears to have been tight direct diaphragmatic closure, which reduced the space in which the spleen and stomach are normally located beneath the left diaphragm. As a result, gastric volvulus was more likely to occur. Gastropexy is necessary for prolonged gastric volvulus. Further studies are required to establish a comprehensive therapeutic strategy for this disease.

## Data Availability

The dataset supporting the conclusion of this article is included within the article.

## Abbreviation

CDH: Congenital diaphragmatic hernia
